# DPP4 and ACE2 in Diabetes and COVID-19: Therapeutic Targets for Cardiovascular Complications?

**DOI:** 10.3389/fphar.2020.01161

**Published:** 2020-08-07

**Authors:** Inés Valencia, Concepción Peiró, Óscar Lorenzo, Carlos F. Sánchez-Ferrer, Jürgen Eckel, Tania Romacho

**Affiliations:** ^1^Vascular Pharmacology and Metabolism Group (FARMAVASM), Department of Pharmacology, School of Medicine, Universidad Autónoma de Madrid, Madrid, Spain; ^2^Instituto de Investigaciones Sanitarias del Hospital Universitario La Paz (IdiPAZ), Madrid, Spain; ^3^Laboratory of Vascular Pathology and Diabetes, FIIS-Fundación Jiménez Díaz, Universidad Autónoma Madrid, Madrid, Spain; ^4^Spanish Biomedical Research Centre in Diabetes and Associated Metabolic Disorders (CIBERDEM) Network, Madrid, Spain; ^5^German Diabetes Center, Institute for Clinical Diabetology, Leibniz Center for Diabetes Research at Heinrich Heine University Düsseldorf, Düsseldorf, Germany

**Keywords:** COVID-19, SARS-CoV-2, diabetes, cardiovascular disease, angiotensin converting enzyme 2, dipeptidyl peptidase 4, gliptins, ACEi

## Abstract

COVID-19 outbreak, caused by severe acute respiratory syndrome (SARS)-CoV-2 coronavirus has become an urgent health and economic challenge. Diabetes is a risk factor for severity and mortality of COVID-19. Recent studies support that COVID-19 has effects beyond the respiratory tract, with vascular complications arising as relevant factors worsening its prognosis, then making patients with previous vascular disease more prone to severity or fatal outcome. Angiotensin-II converting enzime-2 (ACE2) has been proposed as preferred receptor for SARS-CoV-2 host infection, yet specific proteins participating in the virus entry are not fully known. SARS-CoV-2 might use other co-receptor or auxiliary proteins allowing virus infection. *In silico* experiments proposed that SARS-CoV-2 might bind dipeptidyl peptidase 4 (DPP4/CD26), which was established previously as receptor for MERS-CoV. The renin–angiotensin–aldosterone system (RAAS) component ACE2 and DPP4 are proteins dysregulated in diabetes. Imbalance of the RAAS and direct effect of soluble DPP4 exert deleterious vascular effects. We hypothesize that diabetic patients might be more affected by COVID-19 due to increased presence ACE2 and DPP4 mediating infection and contributing to a compromised vasculature. Here, we discuss the role of ACE2 and DPP4 as relevant factors linking the risk of SARS-CoV-2 infection and severity of COVID-19 in diabetic patients and present an outlook on therapeutic potential of current drugs targeted against RAAS and DPP4 to treat or prevent COVID-19-derived vascular complications. Diabetes affects more than 400 million people worldwide, thus better understanding of how they are affected by COVID-19 holds an important benefit to fight against this disease with pandemic proportions.

**Graphical Abstract:**
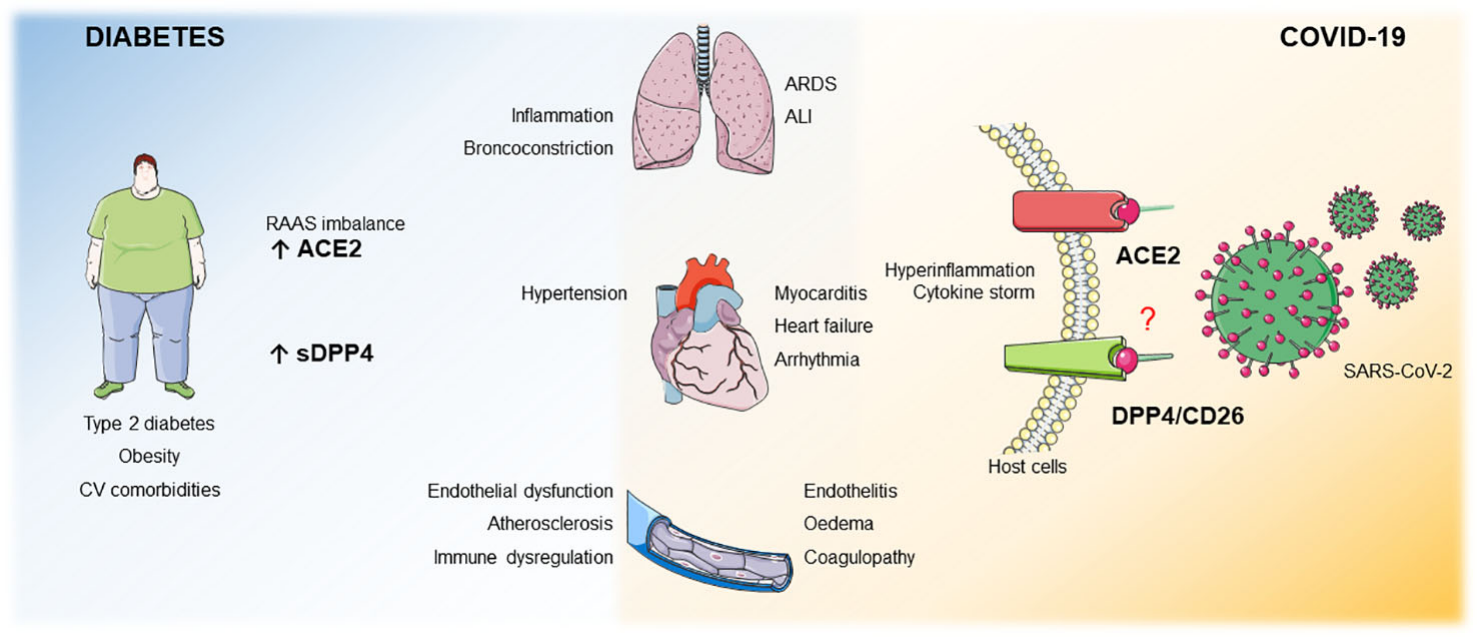
Patients with obesity, T2DM or CV comorbidities display an imbalance in the RAAS system and upregulated sDPP4 levels. These 2 factors *per se* increase the risk for bronchoconstriction, lung inflammation, heart failure, endothelial dysfunction, atherosclerosis and immune dysregulation. ACE2 is a receptor for SARS-CoV-2 and DPP4 has been poposed as potential co/receptor. We hypothesize that in patients with these comorbidities, where both ACE2 and sDPP4 are enhanced, infection with SARS-CoV-2 may result in increased COVID-19 severity with pulmonar complications such as ALI, ARDS and CV complications such as heart failure, arrhythmia, myocarditis, endothelitis, oedema and coagulopathy.

## Introduction

### Diabetes as Promoter of Severity and Mortality in COVID-19

The recent outbreak of COVID-19 pneumonia in China (Guan et al., 2020; Huang et al., 2020) has become an urgent health and economic challenge due to its pandemic proportions. Therefore, there is a current race for developing strategies to treat or prevent COVID-19 infection. COVID-19 is caused by the new severe acute respiratory syndrome coronavirus 2 (SARS-CoV-2). During the last 20 years other two betacoronaviruses bursted, namely SARS-CoV and middle east respiratory syndrome (MERS)-CoV, yet without such pandemic impact.

Similarly to former influenza infections, there is increasing evidence that diabetes is an important risk factor for the severity and mortality of COVID-19 (Hill et al., 2020; Klonoff and Umpierrez, 2020; Zhou et al., 2020). A meta-analysis pointed towards hypertension and cardiovascular (CV) disease and diabetes as the most prevalent cardiometabolic comorbidities in COVID-19 hospitalized patients (Li B. et al., 2020). The first large cohort of hospitalized patients with COVID-19 in Europe confirmed arterial hypertension, followed by chronic heart disease and diabetes as the main comorbidities upon hospitalisation. While at intensive care unit (ICU) the most frequent comorbidities were hypertension, obesity and diabetes (Borobia, 2020; Vaduganathan et al., 2020). Furthermore, recent studies suggest that obesity may be related to increased COVID-19 severity (Petrilli et al., 2020; Simonnet et al., 2020) even in younger patients (Lighter et al., 2020).

Being CV diseases the main cause of morbi-mortality in type 2 diabetic patients, it is not surprising that besides being comorbidities, obesity, type 2 diabetes mellitus (T2DM) and CV diseases have been suggested as risk factors for severity in COVID-19 in several meta-analysis (Wang B. et al., 2020; Zhang D. et al., 2020). In fact, in a retrospective cohort study including 72.314 COVID-19 patients, those with previous CV comorbidities displayed five-fold higher mortality risk (Wu and McGoogan, 2020).

Since initial epidemiological data pointed towards COVID-19 specially affecting older patients where diabetes, CV diseases and obesity are frequent comorbidities, it is under discussion whether these comorbidities and especially diabetes actually increase the risk of infection or only the severity or if they are just co-existing preconditions more frequently found in the severely affected patients (Ceriello et al., 2020; Fadini et al., 2020).

### Pathophysiology of COVID-19: Pulmonar and Cardiovascular Complications

Acute respiratory distress syndrome (ARDS) is the main death cause of COVID-19 (Huang et al., 2020). Autopsy and radiography findings detected acute lung inflammation in patients, characterized by accumulation of infiltrated leukocytes (Li L. et al., 2020). In fact, one of the main causes for ARDS is the cytokine storm, that results in an excessive inflammatory response. Although macrophages do not allow SARS-CoV replication, interaction of SARS-CoV with these cells leads to overproduction of pro‐inflammatory cytokines potentially leading to acute lung injury (ALI) and ARDS (He et al., 2006). Besides macrophages, the role of activated endothelial cells is increasingly acknowledged to be implicated in the inflammatory response in ARDS (Villar et al., 2019), as alveolar–capillary barrier disruption is the key determinant for the progression of severe hypoxemia shown in COVID-19 patients (Li L. et al., 2020). Several epidemiological studies suggest that type 2 diabetic patients display increased risk of developing pulmonary hypertension and worse survival (Kolahian et al., 2019).

Although COVID-19 is primarily acknowledged as a respiratory disease, lung damage can be attributable to a great extent to an initial CV cause such as embolism or pulmonary hypertension. Not only CV complications can be a key cause of death in COVID-19, but those patients with previous CV diseases are at increased risk of death (Guo et al., 2020; Zhou et al., 2020).

In fact, CV impairment manifestations are arising as relevant secondary complications during SARS-CoV-2 infection. On one hand, it has been observed that previous CV conditions worsen upon COVID-19 diagnosis (Guo et al., 2020), although cerebrovascular accidents have intriguingly been also reported in healthy young patients with no history of chronic conditions (Oxley et al., 2020).

The main CV complications reported in COVID-19 are myocardial infarction, arrhythmia, stroke and arterial and venous thromboembolism, including pulmonary embolism (E S Cardiology, 2020; Wang D. et al., 2020). The ability of SARS-CoV-2 to directly infect other cells beyond the lung epithelium causing myocarditis and endothelitis might be the underlying explanation, but also an exacerbated immune and/or coagulation response. SARS-CoV-2 can induce, as other SARS-CoVs, direct damage on the cardiomyocytes, in line to reported interstitial monoculear inflammatory cells infiltration in the myocardium of COVID-19 positive patients (Xu et al., 2020). In fact, cardiac injury has been reported in about 20% of the analysed patients (Guo et al., 2020; Zhou et al., 2020).

A retrospective case series analysing 187 patients in Wuhan observed that patients with underlying CV disease were the ones presenting more severe heart damage, as indicated by elevated troponin T (TnT) and N-terminal pro-brain natriuretic peptide (NT-pro-BNP) levels (Chen et al., 2020; Guo et al., 2020). Indeed, high NT-pro-BNP levels in critially ill patients was also associated with acute respiratory symptoms and increased inflammatory status (Yang C. et al., 2020). Equally, patients with no preexisting heart conditions have shown signs of cardiac damage (Li B. et al., 2020).

Endothelitis was also evidenced by postmortem electron analysis of viral inclusion structures in renal endothelial cells (Varga et al., 2020). In fact, SARS-CoV-2 was able to directly infect engineered human blood vessel organoids *in vitro* (Monteil et al., 2020). Accumulated inflammatory cells and apoptotic bodies near vascular beds (Varga et al., 2020) lead to altered vascular permeability (E S Cardiology, 2020), an important sign of endothelial dysfunction. Endothelial dysfunction is a hallmark of CV disease found in hypertension, diabetes and obesity. However, SARS-CoV-2 might impair endothelium functionality not only on those patients with previous weakened vasculature, but also in healthy individuals. There are case-reports of patients under 50 presenting sudden strokes even with mild manifestations of COVID-19 onset (Oxley et al., 2020).

Coagulopathy has been diagnosed in several fatal and non-fatal cases, with a number of thrombotic parameters altered in COVID-19 patients, including anti-thrombin activity (AT), pro-thrombin time (PT) and D-dimer (Wang D. et al., 2020; Zhang D. et al., 2020). Reported higher D-dimer levels paralleled massive elevation of pro-coagulant von Willebrand factor and factor VIII, indicating endothelial activation (Escher et al., 2020). Endothelial activation leads to chemokine and cytokine release resulting in recruitment of immune cells, perpetuating and spreading inflammation to neighbouring tissues. In fact, a retrospective analysis of the coagulation features in 183 patients determined that fatal COVID-19 cases displayed disseminated intravascular coagulation (Tang et al., 2020), which is strictly linked to endothelial dysfunction and reinforces the sense of SARS-CoV-2 deteriorating endothelium in the lung and beyond.

Thus, CV complications might represent cause and effect of pulmonary manifestations of COVID-19. Microvascular dysfunction of lung capillaries implies the development of pulmonary oedema due to increased vascular permeability, which also allows SARS-CoV-2 to invade other vascular beds (Varga et al., 2020). In the same way, clots coming from peripheral vessels can migrate to the lungs or even the brain and cause pulmonary thromboembolism or strokes. In fact, evidence in both animals and humans has demonstrated that fibrinolytic therapy in ARDS and ALI improves survival (Wang J. et al., 2020). Therefore, vascular function preservation in COVID-19 treatment, and more specifically, in diabetic patients might improve the disease prognosis.

Angiotensin-converting enzyme-2 (ACE2) is a pivotal protein in the protecting branch of the renin-angiotensin-aldosterone system (RAAS), as it metabolizes angiotensin (Ang) II into its physiological antagonist Ang-(1-7), which oposses its detrimental vascular effects (Santos et al., 2018). In fact, type 2 diabetic patients display an umbalanced AngII/Ang-(1-7) ratio (Srivastava et al., 2019). Analogously, DPP4 upregulation has been associated with vascular pathophysiology in diabetes and obesity (Röhrborn et al., 2015; Wronkowitz et al., 2015). We hypothesize that enhanced circulating levels of soluble DPP4 and misbalanced ACE2 expression found in obesity and T2DM may contribute to the severity of COVID-19 related to these disease/comorbidities. Furthermore, due to their vascular effects, both DPP4 and ACE2/Ang II axis arise as potential therapeutic targets to ARDS and CV complications in COVID-19 that might be pharmacologically scoped, as it will be detailed below.

## Mechanisms of SARS-CoV-2 Entry Into Host Cells

Similarly to SARS-CoV, it has been proposed that SARS-CoV-2 enters human cells using ACE2 as receptor (Zhang H. et al., 2020). Once attached to ACE2 *via* the receptor binding domain (RBD) in the viral spike protein, it is primed by the host serine protease TMPRSS2, which ultimately allows fusion of viral and cellular membranes. Although ACE2 is ubiquosly expressed, with higher expression in the CV system, within the lung it is specifically found in alveolar epithelial cells and lung endothelial cells (Santos et al., 2018).

In the lung, SARS-CoV infects mainly pneumocytes and macrophages (Shieh et al., 2005). The peptidases in the lung epithelium and endothelium have been proposed as primary sites for coronavirus infection once the viral infection progresses in the respiratory tract. In this line, it has been suggested that the use of peptidases by coronaviruses to enter host cells might rely more on their abundance than on their proteolytic activity (Li et al., 2003). However, the specific proteins that facilitate SARS-CoV-2 entry in host cells remain unclear.

It has been suggested that other betacoronavirures require the participation of diverse surface proteins for human cells invasion. Thus, sialic acid receptors and C-type lectin receptor CD209/DC-SIGN, expressed on the surface of macrophages and dendritic cells, participate in SARS-CoV invasion into immune cells *in vivo* (Li et al., 2003; Kuba et al., 2005). *In vitro* the transmembrane glycoprotein CD147/basigin has been described to mediate SARS-CoV invasion. Interestingly, the interaction of CD147 and SARS-CoV-2 RBD has been observed and successfully prevented by competitive anti-CD147 treatment (Wang K. et al., 2020).

On the other hand, MERS-CoV binds to human DPP4/CD26 to infect host cells (Raj et al., 2013). A very recent study predicting the structure of the SARS-CoV-2 spike glycoprotein and its glycan shield pattern suggests that DPP4/CD26 may also act as receptor for SARS-CoV-2 (Vankadari and Wilce, 2020). In favor of this hypothesis lays the highest co-expression of DPP4 with ACE2 (Qi et al., 2020). In fact, unique residues of SARS-CoV-2 glycoprotein predicted to bind ACE2 can also interact with DPP4 (Vankadari and Wilce, 2020).

Since MERS-CoV binds to DPP4, a transgenic mouse model expressing human DPP4 on relevant cells as epithelial and alveolar pulmonary cells has been used to study the impact of diabetes on MERS-CoV infection. MERS-CoV-infected diabetic mice expressing DPP4 suffered more severe symptoms for longer time than corresponding infected controls (Kulcsar et al., 2019). Therefore, we hypothesize that DPP4 might represent a link between diabetes and morbi-mortality/severity of COVID-19.

## RAAS and sDPP4 in Diabetes and Other Comorbidities

### Cardiovascular Effects of ACE-Ang II-AT1R Axis Upregulation and Ang-(1-7)/Mas Axis Dowregulation

**Figure 1:**
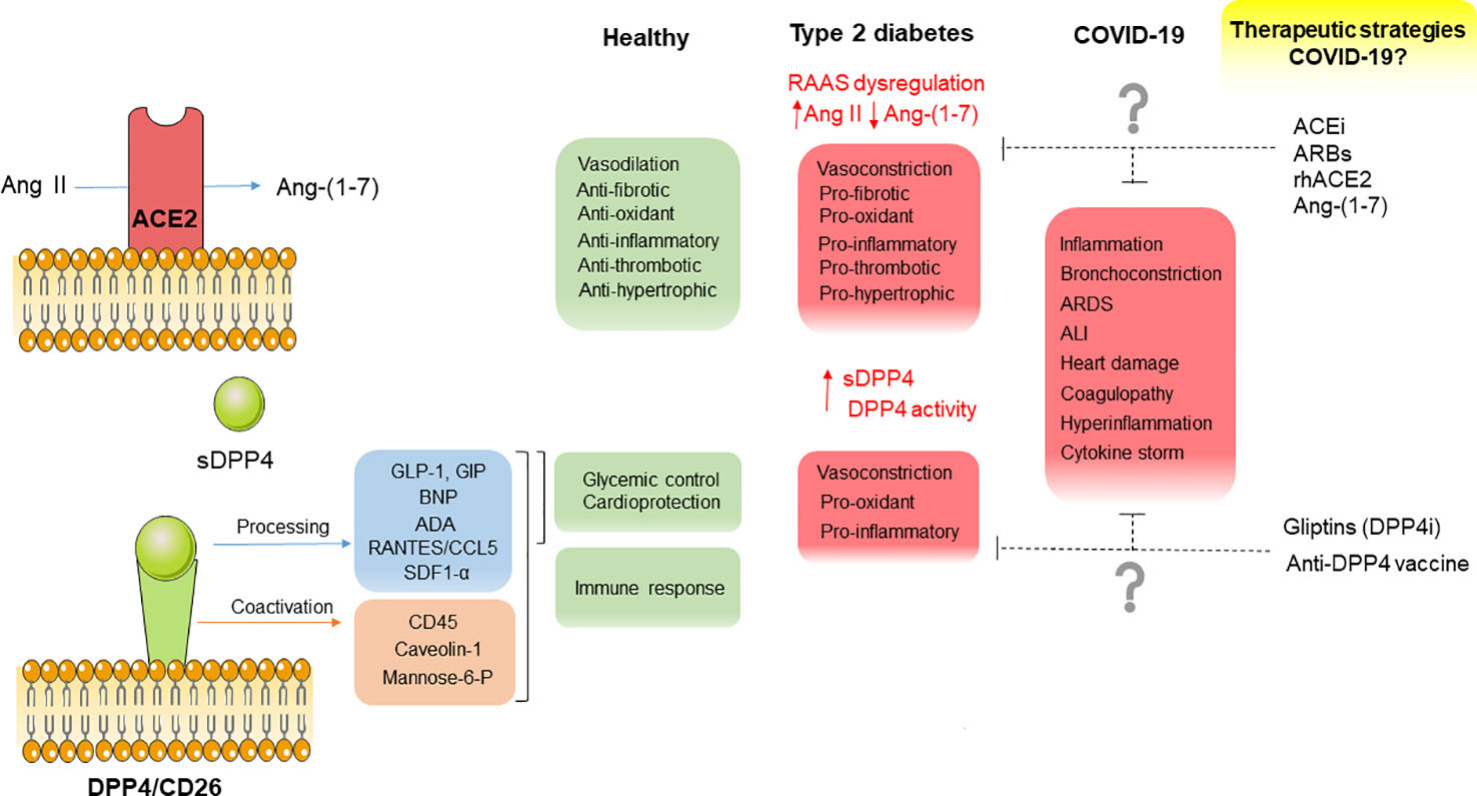
Diagram summarizing the main physio- and pathophysiological functions of ACE2 and DPP4, their potential contribution to CV complications in COVID-19 as well as potential therapeutic strategies interfering with these 2 factors to counteract CV complications in COVID-19 and type 2 diabetic patients.

The RAAS is a key player in the regulation of CV homeostasis in both health and disease. Ang II is the primary effector of RAAS, upregulated in the context of vascular pathophysiology exerting vasoconstrictor, pro-oxidant, pro-inflammatory, pro-fibrotic and proliferative effects, mostly through AT1 receptors (Romero et al., 2019). ACE2 is a pivotal protein in the protecting branch of RAAS, as it metabolizes Ang II into Ang-(1-7) and Ang I into Ang-(1-9) which is in turn metabolized as Ang-(1-7) by angiotensin-converting enzyme (ACE). Ang-(1-7) opposes to Ang II-induced detrimental vascular effects participating in vasodilator, anti-inflammatory, anti-oxidant, anti-proliferative, anti-thrombotic and anti-senescence responses through Mas receptors (Santos et al., 2018; Romero et al., 2019). Moreover, Ang-(1-7) stimulates pleiotropic cytoprotective pathways (Nrf2-HO-1 axis) (Romero et al., 2019) (**Figure 1**).

Under physiological conditions, the RAAS peptides are produced in a balanced manner, whereas misbalance of the RAAS has been implicated in arterial hypertension and diabetes. It has been proposed that increased Ang II can contribute to hyperglycaemia and dyslipidaemia, impairment of vascular function and inflammation. ACE2 deficiency increased vascular inflammation and atherosclerosis in ApoE knockout mice (Thomas et al., 2010). In fact, ACE2-Ang-(1-7)/Mas axis was shown to participate in the reduction of obesity-induced inflammation and chronic renal failure (Santos et al., 2018). In addition, Ang-(1-7) can exert anti-inflammatory effects over RAAS-independent pro-inflammatory stressors as interleukin (IL)-1β (Villalobos et al., 2016; Romero et al., 2019).

SARS-CoV spike glycoprotein injection into mice decreased ACE2 expression levels, worsening lung injury (Kuba et al., 2005). Similarly, SARS-CoV-2-ACE2 binding induced ACE2 internalization (Zhang H. et al., 2020), resulting in Ang II accumulation and insufficient Ang-(1-7) synthesis. Importantly, ACE2 is also directly down-regulated by pro-inflammatory cytokines (Lang et al., 2006). ACE2 serves both as entry receptor and protective mediator in the lung (Monteil et al., 2020), in concordance with data observing decreased ACE2 expression and activity in human idiopathic pulmonary fibrosis (Li et al., 2008). Taking that ACE2 is highly expressed in the endothelium and heart (Santos et al., 2018), decreased ACE2 levels may also lead to imbalanced RAAS signalling and potential disruption of CV homeostasis in COVID-19 patients.

### Cardiovascular Effects of sDPP4 Upregulation

DPP4/CD26 is a serine protease cleaving a wide variety of substrates including the incretin hormones glucagon-like peptide 1 (GLP-1) and gastric inhibitory peptide (GIP), cytokines and growth factors (Röhrborn et al., 2015). DPP4 can be found as a membrane-bound form or be shedded as a soluble form (sDPP4) that maintains its enzymatic activity (Röhrborn et al., 2015). We hypothesize that enhanced circulating sDPP4 may promote T2DM-related severity of COVID-19.

In the context of obesity and T2DM, both AT and liver have been proposed as relevant sources of sDPP4 in humans, although the main source remains under discussion. Our group identified sDPP4 as a novel adipose-derived factor (Lamers et al., 2011), whose circulating levels are enhanced in visceral fat from obese and insulin-resistant patients and correlate with BMI (Sell et al., 2013). While sDPP4 plasma activity and levels have been associated with liver apoptosis, fibrosis, fat content, and NAFLD (Baumeier et al., 2017; Romacho et al., 2020).

DPP4 can promote local and systemic inflammation through its immunomodulatory activity. Thus, DPP4 triggers T cell activation, proliferation and cytokine production alone (Focosi et al., 2020) or through the interaction with immune partners on antigen presenting cells as CD45, caveolin-1, manose-6 phosphate receptor, or adenosine deaminase (ADA) (Röhrborn et al., 2015; Williams et al., 2015) (**Figure 1**). DPP4 expression is enhanced on blood T lymphocytes from type 2 diabetic patients and correlates with insulin resistance and glycated hemoglobin (Lee et al., 2013). sDPP4 treatment enhanced LPS-induced tumor necrosis factor (TNF)-α and IL-6 secretion in activated monocytes. Besides its upregulation in T2DM and obesity, DPP4 expression is increased in senescent cells (Kim et al., 2017).

Immunomodulatory effects of DPP4 have been also demonstrated in animal models. Exogenous injected sDPP4 increased monocyte migration *in vivo* in a model of low-density-lipoprotein receptor-deficient mice. Interestingly, upregulation of DPP4 in obese and diabetic animals have evidenced also its role on immune responses dysregulation. In this regard, genetic depletion of DPP4 improved adipose tissue inflammation in dark agouti rats (Röhrborn et al., 2015; Romacho et al., 2020).

Some DPP4 substrates with CV impact are upregulated in obesity and/or T2DM as well as in COVID-19 such as CXCL5/RANTES and BNP (Niraula et al., 2018; Adela et al., 2019). In fact, high BNP levels were positively correlated with the incidence of CV events and mortality in HIV-infected patients, and was suggested as an indicator of patients condition deterioration from mild to severe prognosis also in COVID-19 (Yang C. et al., 2020).

DPP4 is also known to interact with extracellular matrix (ECM) proteins, participating in both cell migration and tissue remodelling. By binding to fibronectin, DPP4 can promote T cell helper migration and accummulation in areas with increased ECM proteins, such as damaged blood vessels (Aroor et al., 2013). DPP4 also interacts with fibroblast activation protein (FAP)-α/seprase leading to migration and invasion of endothelial cells into collagen matrices (Ghersi et al., 2006). Moreover, stromal cell-derived factor alpha (SDF-1α) is also a substrate of DPP4, and inhibition of DPP4 has been observed to increase SDF-1α/CXCR4-induced mobilization of endothelial cells from bone marrow to injured vascular sites (Aroor et al., 2013).

Via its interaction with ADA, DPP4 activates plasminogen-2 leading to increased plasmin levels which contributes to degradation of ECM proteins and the activation of matrix metalloproteinase (MMP)-4, which facilitates immune cells migration and diapedesis (Kameoka et al., 1993; Röhrborn et al., 2015). It has been recently proposed that plasmin among other proteases may cleave a new furin site in the spike protein of SARS-CoV-2, leading to an increase in infectivity and severity. This excess of plasmin may contribute to the hyperfibrionolysis resulting in enhanced D-dimer in severe patients (Ji et al., 2020).

Importantly, besides its immunomodulatory effects, sDPP4 can exert direct deleterious effects on the vascular wall. sDPP4 directly promoted human smooth muscle cell (hVSMC) proliferation and inflammation *via* NF-κB activation resulting in the upregulation of pro-inflammatory cytokines such as monocyte chemoattractant protein (MCP)-1, IL-6 and IL-8, through a novel mechanism mediated by the activation of the protease activated receptor (PAR)-2 (Wronkowitz et al., 2014). DPP4 is itself expressed in the vasculature (Zhong et al., 2015). In hVSMC DPP4 shedding is stimulated by hypoxia in parallel to MMP-1 expression (Röhrborn et al., 2014). Moreover, PAR2 activation induced by sDPP-4 triggered endothelial dysfunction in mesenteric microvessels through thromboxane A2 release mediated by cyclooxygenase (COX) and the thromboxane A2 receptor (TP) activation (Romacho et al., 2016).

Thus we hypothesize that DPP4 expression may contribute to T2DM-related severity of SARS-CoV-2 infection. Furthermore, due to its vascular effects, sDPP4 arises as a potential contributor to ARDS severity by potentially inducing inflammation and bronchoconstriction.

## Therapeutic Potential of RAAS-Targeted Drugs in Diabetic Patients With COVID-19

### ACEis and ARBs

Despite the initial concerns about ACE inhibitors (ACEi) and AT1 receptor blockers (ARB) treatment potentially predisposing patients to SARS-CoV-2 infection and/or increased COVID-19 severity, the European Medicines Agency advised to continue those prescriptions (EMA/143324/2020; EMA/284513/2020). ACEi/ARBS are primarily prescribed for treating hypertension and associated left ventricular dysfunction, employed in about 35% of the hypertensive population in China (Vaduganathan et al., 2020).

SARS-CoV-2-ACE2 binding leads to ACE2 downregulation and concomitant misbalance of Ang II/Ang-(1-7) ratio towards Ang II-induced detrimental effects. Ang II binding to AT1 receptors increases pulmonary vascular permeability contributing to lung pathology, as previously shown in relation to SARS-CoV lethality (Kuba et al., 2005). Some reports observed that ACEi fail to inhibit ACE2 (Turner et al., 2002) and suggested that small peptide specific ACE2 inhibitors as DX600 as more efficient inhibiting SARS-CoV cell entry *in vitro* (Guy et al., 2005). Nevertheless, ACEi or AT1R may induce ACE2 upregulation as detected both in animal models at mRNA and protein level (Sukumaran et al., 2011), as well as urinary excretion in olmesartan-treated hypertensive patients (Furuhashi et al., 2015). Taking that increased ACE2 expression also leads to increased Ang-(1-7) production, chronic ACEi and ARB may protect COVID-19 patients. A former study showed that ARB prevented ALI damage in SARS-CoV-infected mice (Kuba et al., 2005). Accordingly, previous studies have shown that ACEi treatment is associated with a diminished risk of pneumonia in both type 1 and type 2 diabetic patients (van de Garde et al., 2007).

In this line, recent studies have demonstrated that the risk of severe COVID-19 was decreased in patients taking ARB in comparison to no-hypertensive patients (Lu et al., 2020; Meng et al., 2020). Interestingly, ACEi/ARB-treated COVID-19 positive patients displayed lower concentrations of CRP and procalcitonin (Yang G. et al., 2020), suggesting that the benefit of ACEi and ARB might rely on their anti-inflammatory effects. Indeed, ARB treatment in rats reduced plasma TNF-α, IL-1β and IL-6 levels and increase IL-10 (Simões e Silva et al., 2013). The ongoing clinical trial BRACE-CORONA (NCT04364893) will compare the impact of temporary discontinuation of ACEi or ARB on mortality and days of hospital stay on patients with SARS-CoV-2 infection (Lopes et al., 2020).

### Ang-(1-7) Analogues

Restoring the balance between the RAAS peptides may be an efficient pharmacological approach against COVID-19. The loss of protective Ang-(1-7) as a result of reduced ACE2 availability, may exaggerate the hyper-inflammatory environment in the lung and beyond. Ang-(1-7) supplementation may reveal as an effective pharmacological strategy to attenuate severe CV complications in COVID-19, as we have recently hypothesized (Peiró and Moncada, 2020). In animal models of ALI and ARDS, Ang-(1-7) has demonstrated anti-inflammatory and anti-fibrotic effects via, at least in part, the G protein-coupled receptor Mas (Klein et al., 2013; Santos et al., 2018). Accordingly, administration of Ang-(1-7) and its synthetic analogue AVE 00991 have been reported to reduce leukocyte adhesion to the microvascular endothelium in a model of arthritis (Simões e Silva et al., 2013).

Indeed, the COVID-ARA2 (NCT04337190) and ATCO clinical trial (NCT04332666) will evaluate Ang II blockade and Ang-(1-7) supplementation in the context of lung disease in COVID-19 patients.

### rhACE2 as Decoy Factor

It has been demonstrated that recombinant human ACE2 (rhACE2), already tested in phase 2 clinical trials for ARDS/ALI treatment (NCT01597635), blocks SARS-CoV-2 infection in Vero cells and binding to both human blood vessels and human kidney organoids (Monteil et al., 2020). Indeed, the recombinant sACE2 drug will be used in an interventional clinical trial over 200 COVID-19 patients (APN01-COVID-19, NCT04335136).

However, rhACE2 has been suggested not only to hamper SARS-CoV-2 entry into host cells but also potentially to protect from lung injury and vascular dysfunction. ACE2 administration lowered blood pressure and improved endothelial function in an Ang-(1-7)-mediated manner in a model of spontaneously hypertensive stroke-prone rats (SHSPR) (Focosi et al., 2020). In this line, direct activation of ACE2 by NCP-2454 improved pulmonary arterial compliance in a model of pulmonary hypertension (Haga et al., 2015). Moreover, administration of rhACE2 normalized Ang II levels in isolated human hearts with dilated cardiomyopathy (Basu et al., 2017).

## Therapeutic Potential of Targeting DPP4 in Diabetic Patients With COVID-19

### Gliptins

During COVID-19 infection vascular functionality is endangered. Therefore, preservation of vascular integrity might be a key priority. In case DPP4 represents a link between diabetes and the severity of COVID-19, DPP4i may hold additional therapeutic value to protect diabetic patients suffering COVID-19, as recently especulated (Drucker, 2020; Iacobellis, 2020).

DPP4i, the so-called gliptins, are antidiabetic drugs based on the control of glucose homeostasis through inhibition of DPP4 enzymatic activity. DPP4 cleaves and inactivates the incretin hormones GLP-1 and GIP, which account for up to 60–70% of postprandial insulin release. This way DPP4i prolong the half life of incretins. Besides its action on incretins, gliptins have been proposed to exert other off-target effects including CV effects. Regarding DPP4 inhibition as a therapeutic strategy to treat and prevent CV diseases in obese and/or type 2 diabetic patients there is still an ongoing discussion. Importantly, gliptins are preferred as add-on therapy in individuals with diabetes coursing with previous chronic kidney disease or additional CV disease (Hanssen and Jandeleit-Dahm, 2019).

CV safety of gliptins has been confirmed in several CV outcome trials (SAVOR TIMI 53, NCT01107886; EXAMINE, NCT00968708; TECOS, NCT00790205; CARMELINA, NCT01897532). In relation to their glycemic action, gliptins have demonstrated no risk of hypoglycemic episodes, neutral influence on body weight and a significant reduction of glycated hemoglobin levels (HbA1c), which are important factors associated with reduced CV risk and mortality (Zoungas et al., 2010). Moreover, DPP4i have positive effects over surrogate vascular endpoints, namely blood pressure, lipemia and endothelial function, albeit these CV outcome trials failed in demonstrating additional benefits of gliptins on major CV events (Scheen, 2018). However, gliptins are widely used as T2DM treatment. It has been shown that combined metfomin and gliptin therapy significantly decreased the relative risk of non-fatal CV events, CV mortality and all-cause mortality, in comparison to other oral antihyperglycemic drugs as sulfonylureas (Wang et al., 2017).

Preclinical studies have shed light on the mechanisms by which gliptins might improve CV functionality. Increased bioavailability of substrates of DPP4 such as GLP-1 confers indirect cardioprotective effects to gliptins. Beyond GLP-1, DPP4 inhibition increases the effects of other substrates with cardiovascular impact. T2DM patients trated with sitagliptin displayed higher SDF-1α plasma levels resulting in increased endothelial progenitor cells (EPCs), which play a key role in vascular repair (Fadini et al., 2010). *In vitro*, SDF-1α was also able to improve blood flow in a model of peripheral artery disease (Segers et al., 2011). Equally, an increase in BNP improved the regulation of natriuresis and the vasodilatory responses (Brandt et al., 2006).

In our hands, both hVSMC proliferation and inflammation as well as *ex vivo* endothelial dysfunction exerted by sDPP4 were equally prevented by the experimental and clinically available DPP4i K579 and linagliptin, respectively (Wronkowitz et al., 2014; Romacho et al., 2016) That suggests that these drugs may add CV benefit beyond regulating glucose homeostasis and in a GLP-1-independent manner.

In animal models of diabetes, gliptins have also proven protective effects. Vildagliptin-treated streptozotocin (STZ)-induced diabetic rats presented reduced oxydative stress and ICAM-1 and plasminogen activator inhibitor type-1 (PAI-1) expression (Maeda et al., 2012). In db/db mice linagliptin improved cardiac dysfunction by reducing the activation of the Nlrp3/ASC inflammasome and the upregulation of collagen-1 and collagen-3 (Birnbaum et al., 2019). Similarly, sitagliptin also improved cardiac function in mice (Picatoste et al., 2013). Anti-oxidant properties of DPP4i have been also shown under acute inflammation in a mouse model of LPS-induced sepsis (Beckers et al., 2017).

In type 2 diabetic patients, sitagliptin treatment reduced the molecular markers of inflammation as CRP and IL-6 in mononuclear cells (Makdissi et al., 2012), as well as circulating levels of TNF-α, IL-1β, IL-6, intracellular adhesion molecules and CRP (Tremblay et al., 2014). Sitagliptin also impoved the flow-mediated vasodilation in diabetic adults (Barchetta et al., 2019). Importantly, sitaglipitin conferred cardioprotection in diabetic patients with chronic kidney disease (CKD) reducing the Ang II/Ang-(1-7) ratio (Beraldo et al., 2019).

Gliptins can preserve endothelial function by their reported anti-inflammatory, anti-oxidant and potentially protective effects on the vascular system (Avogaro and Fadini, 2018), which are beneficial aspects in the fight against COVID-19. Additionally, a meta-analysis of large randomized clinical trials demonstrated that gliptin treatment did not increase the risk of general infections in type 2 diabetic patients (Yang et al., 2016).

Besides offering potential CV protection, gliptins might be also employed to restrain SARS-CoV-2 binding to host cells. DPP4i ability to hamper coronavirus entry to host cells has been studied. Sitagliptin, vildagliptin or saxagliptin could not block MERS-CoV access to Vero cells (Raj et al., 2013). Contrarily, sitagliptin was able to hamper MERS-CoV infection in macrophages by reversing the immunosuppressive capacity of the virus (Al-Qahtani et al., 2017).

All approved gliptins are competitive reversible inhibitors of DPP4 enzymatic activity (Deacon, 2011). However, gliptins can be categorized in 3 classes based on the subsites within the DPP4 molecule they occupy. Class 1 comprises peptidomimetic DPP4i with the most basic binding to DPP4 such as vildagliptin and saxagliptin. While class 2 inhibitors (alogliptin and linagliptin) and class 3 inhibitors (sitagliptin and teneligliptin) display an increased number of interacting points. The more interacting points the higher inhibitory potency (Nabeno et al., 2013). DPP4 extracelullar structure consists of two domains comprised of a eight γ-bladded β-propeller domain and a C-terminal α,β-hydrolase domain with the catallytic triad (Asp708, His740, Ser630) (Röhrborn et al., 2015). Conserved aminoacids on DPP4 catalytic site are displayed in pockets S1 and S2. However, the catalytic pocket does not colocalize with the proposed binding sites of SARS-CoV-2 to DPP4 (Vankadari and Wilce, 2020). Nevertheless, previous structural studies regarding MERS-CoV-DPP4 interaction demonstrated that residues in the β-propeller domain of DPP4 (Arg54–Asn497) were essential for virus S1 glycoprotein binding, and some of them are also shared by SARS-CoV-2 (Vankadari and Wilce, 2020).

The residue Asp542 was ascertained as a binding point of SARS-CoV-2 to DPP4, as it was also demonstrated for MERS-CoV (Vankadari and Wilce, 2020). Interestingly, class 1 and 2 gliptins (saxagliptin, alogiptin and linagliptin) are able to bind near this residue (Tyr547) in a zone beyond DPP4 pockets S1 and S2 called S1’ subsite (Nabeno et al., 2013). Uracil ring of alogliptin and linagliptin cause a conformational change on DPP4 S1’ subsite hampering its enzymatic activity (Nabeno et al., 2013). Therefore, this type of xhantine-based non-peptidomimetic gliptins could potentially impair SARS-CoV-2 binding to one of its preferred sites in DPP4. In addition, linagliptin, and to a lesser extent vildagliptin, have been predicted to bind ACE2 with same or even better binding energy than they have for DPP4 (Abouelkheir and El-Metwally, 2019), suggesting additional benefit as SARS-CoV-2 therapy. Whether if more complex class 3 gliptins could exert a steric hindrance to SARS-CoV-2 acess into DPP4 needs further investigation. In this line, a very recent approved clinical trial SIDIACO (NCT 04365517) will study the clinical response of T2DM patients with COVID-19 treated with sitagliptin.

DPP4 immunomodulatory function raises the question whether DPP4i might alter the immune response in diabetic patients (Shao et al., 2020). The pharmakokinetic and pharmacodynamic properties of DPP4i allow that DPP4 enzymatic activity in tissues and circulation is not completey blocked (Drucker, 2020). Both DPP4 inhibition with des-fluro sitagliptin and genetic deletion of DPP4 in mice did not alter T-cell dependent immune responses (Vora et al., 2009). Sitagliptin, vildagliptin and saxagliptin did not alter innate immune response triggered by Toll-like receptor (TLR) activation in terms of secretion, co-stimulation, T cell proliferation and migration in human cells *in vitro* and in mice *in vivo* (Bergman et al., 2006).

An initial meta-analysis reported that although DPP4i is not associated with an increased risk for respiratory infections they could increase the risk of urinary tract and nasopharyngitis infection (Amori et al., 2007). A more recent meta-analysis assessing the impact of long term use of sitagliptin in T2DM patients determined that there was not an enhanced risk of infection (Gooßen and Gräber, 2012). Another study compared the risk of respiratory tract infections among glucose-lowering therapies and found no increased risk linked to DPP4i in T2DM patients (Gamble et al., 2018). However the potential induction of leucopenia (Pitocco et al., 2010), angioedema (Gosmanov and Fontenot, 2009) or cough in asthma (Baraniuk and Jamieson, 2010) by DPP4i represent potential pitfalls to their use as therapy in COVID-19 (Pitocco et al., 2020). Although 14 years after the approval of DPP4i there have not been major safety issues related to long term use of gliptins. Nevertheless further clinical research will help determine if DPP4i can affect infection risk, T cell development and immune homeostasis in T2DM (Klemann et al., 2016).

### sDPP4 as Soluble Decoy Factor

Exogenous administration of sDPP4 inhibited MERS-CoV infection in VERO cells (Raj et al., 2013). Therefore, in case it is demonstarted that SARS-CoV-2 can bind DPP4, exogenous acute administration of sDPP4 as a decoy factor to compete for binding of the virus to endogenous DPP4 in COVID-19 target tissues could be explored.

sDPP4 load benefit might not only rely on virus trapping but also on the blockade of detrimental pathways affecting/worsening immune responses and importantly, to potentially prevent COVID-19 effects on the vasculature. In this line, exogenous administration of sDPP4 was effective to bind to ADA and prevent the formation of endogenous DPP4/CD26-ADA complex in human dendritic cells/macrophages resulting in impeded activation and proliferation of T-cells *in vitro* (Zhong et al., 2013). Disruption of RANTES/CCL5-CCR5 axis with the anti-CCR5 antibody leronlimab reduced IL-6 plasma leves and plasma viral load in COVID-19 patients (Patterson et al., 2020). It has been proposed that sDPP4 may directly truncate CCL5/RANTES impeding its union to CCR5 (Iwata et al., 1999) therefore sDPP4 may potentially cause the same effect as leronlimab treatment, leading to an improved immune response and reduced cytokine storm.

In the case of diabetic patients where significantly increased sDPP4 plasma concentration are already present (Sell et al., 2013), the potential risk-benefit of exogenous administration of sDPP4 as decoy factor should be carefully investigated.

### Anti-DPP4 Vaccine

Another therapeutic approach proven succcesful to regulate plasma DPP4 activity in mice is anti-DPP4 vaccination. Anti-DPP4 vaccine did not cause any side effect on immune cell activation nor immune-mediated attack towards DPP4-expressing cells or tissues. Furthermore it was able to reach comparable effects to gliptin treatment in regard to glycaemia control and GLP-1 plasma levels in mice models of both type 1 and type 2 diabetes (Pang et al., 2014; Li et al., 2017). In this line, a humanized IgG1 monoclonal antibody (YS110) with high affinity for CD26 was evaluated in a phase I clinical trial, with demonstrated no effect on T-cell proliferation and cytokine production *in vitro* on human CD26-positive lymphocytes (Angevin et al., 2017). Interestingly, YS110 also inhibited MERS-CoV infection (Ohnuma et al., 2013). However, it has been discussed that *in vivo* administration of anti-DPP4 antibodies could neutralize plasma sDPP4 before it can coate cellular DPP4 and impede virus entry. In this situation, intranasal application of sDPP4 or anti-DPP4 antibodies has been presented as a plausible solution to overcome such effects (Xia et al., 2014).

On the other hand, DPP4/CD26-related signaling was successfully blocked by the soluble fusion protein Caveolin-Ig, which demonstrated additional immunosuppresive effects (Ohnuma et al., 2009). Tissue-factor pathway inhibitor (TFPI) is a biological blocker of DPP4. Morever, due to its anticoagulant properties (Mast, 2016), TFPI could be seen as a positive aspect in the context of COVID-19 treatment, albeit potential clinical applications have to be carefully evaluted due to its reported role in cancer (Fei et al., 2017).

## Combined Therapeutic Potential Targeting RAAS and DPP4

DPP4 signalling and RAAS dysregulation bidirectional crosstalk has been proposed previously. Ang II was able to induce DPP4 activity *in vitro* and *in vivo* (Aroor et al., 2016). Equally, DPP4 inhibition counteracted RAAS overestimulation in a model of renal ablated rats, where sitagliptin treatment increased Ang-(1-7) and ACE2 expression and activity and reduced AT1 receptor expression and Ang II levels (Beraldo et al., 2019). Thus it has been proposed that DPP4i can attenuate the progression of CV disease by opposing RAAS negative effects.

ACE2 and DPP4 present common features as endopeptidases even sharing a wide variety of experimental inhibitors (Abouelkheir and El-Metwally, 2019). Hence, the idea of combining inhibitory properties of ACEi and DPP4i against COVID-19 could be appealing. However, it has been observed that DPP4i therapy, when combined with high-dose ACEi, could counter the blood pressure lowering effect of ACEi (Aroor et al., 2013). Combination of ACEi and DPP4i seems unreasonable due to reported production of angioedema upon coadministration (Brown et al., 2009). Angioedema is a potentially fatal adverse reaction as characteristic swelling is generally located around face and neck endangering block of upper airways. Both proteases ACE2 and DPP4 have the capacity to proteolyze bradykinin and substance P leading to accumulation of such vasoactive kinins inducing vasodilatation and increase in vascular permeability (Brown et al., 2009). Contrarily, single ACEi or gliptin-associated angioedema is rare (0.1–0.7%) and in some cases disappeared after changing to a different type in the same drug class.

On the other hand, it has been suggested that combination of ARB and DPP4i holds a more effectve therapy for the management of hypertension, renal function and glycaemic control in T2DM (Aroor et al., 2013). The combination of linagliptin and telmisartan induce sinergistic protective effects in the CV functionality of nephrectomized rats (Beraldo et al., 2019).

Therefore, against SARS-CoV-2 the combination of virus entry blockers (ACEi or DPP4i) and virus capturing decoy factors (rhACE2, sDPP4) would be a better option. However, new perspectives into de design of multitarget drugs holds an important therapeutic oportunity as demonstrated by the synthesis of an enalapril-sitagliptin merged compound, which exhibited dual inhibitiory action against human ACE and DPP4 (Abouelkheir and El-Metwally, 2019), with expected reduced drug-drug interactions and undesirable side effects. Moreover, a milk-derived heptapeptide was found to bind and inhibit both ACE and DPP4 activity (Ashok et al., 2019).

## Conclusion

Obese and type 2 diabetic patients are at higher risk of SARS-CoV-2 severity since both conditions favour CV complications *per se*. Therefore, in the meantime vaccines are being developed and anti-viral drugs are investigated, therapeutic strategies to fight COVID-19-induced CV alterations are urgently needed.

It is well known that in T2DM and the metabolic syndrome there is a counterbalance in the RAAS system. Therefore, strategies aiming to regulate this counterbalance may be a useful pharmacological strategy to prevent endothelial cell activation and lung damage in COVID-19 (**Table 1**). Some studies suggest that ARB and ACEi may also contribute to increase Ang-(1-7) and reduce the risk of severity of COVID-19. Another potential strategy is to provide direct Ang-(1-7) supplementation with analogues to attenuate severe cardiovascular complications in COVID-19 as recently published by us (Peiró and Moncada, 2020). The role of ACE2 as SARS-CoV-2 receptor, may lead to reduced ACE2 availability and potentially resulting in hyperinflammation. The use of rhACE2 as decoy receptor deserves further investigation.

**Table 1:** Summarized effects of RAAS-targeted drugs (ACEi, ARBS, Ang-(1-7) analogues and rhACE2) reported in preclinical (*in vivo* and *ex vivo*) and clinical research.

	Preclinical data		Previous clinical trials	COVID-19
	Cells	Animal models		Ongoing clinical trials
**ACEi** **ARBs**	Block SARS-CoV entry (Guy et al., 2005)	Prevention of ALI in SARS-CoV-infected mice (Kuba et al., 2005) Cardiac protection: ACE2 and Ang-(1-7) increase in autoimmune myocarditis in rats (Sukumaran et al., 2011)	Increased ACE2 and Ang-(1-7) in hypertensive patients (Furuhashi et al., 2015) Reduced risk of pneumonia in type 1 and type 2 diabetic patients (van de Garde et al., 2007)	Diminished risk of severe COVID-19 in ARB-treated patients (Meng et al., 2020) Lower concentrations of CRP and procalcitonin in ACEi/ARB treated patients (Yang G. et al., 2020) ≥ 20 registered clinical trials: CAPTOCOVID (NCT04355429), RAMIC (NCT04366050), NCT04345406 (capto/enalapril), NCT04355936 (telmisartan).
**Ang-(1-7) analogues**	Vasodilatory and cytoprotective effects as anti- inflammatory, -oxidant, -proliferative, -thrombotic, -senescent (Santos et al., 2018; Romero et al., 2019)	Anti-inflammatory and anti-fibrotic effects in ALI and ARDS models (Klein et al., 2013; Santos et al., 2018) Prevention of cardiac fibrosis in SD rats (Grobe et al., 2007) Impaired leukocyte adhesion to microvascular endothelium in arthritis (Simões e Silva et al., 2013)		ATCO (NCT04332666) TXA127 COVID (NCT04401423) NCT04375124 (Ang-(1-7) vaccine)
**rhACE2**	Block SARS-CoV-2 infection in Vero cells, human blood vessels and kidney organoids (Monteil et al., 2020) Recovered Ang II levels in human isolated hearts (Basu et al., 2017)	Blood pressure decrease and improved endothelial function in SHSRP rats (Focosi et al., 2020) Improved pulmonary arterial compliance in pulmonary hypertension (Haga et al., 2015)	Phase II clinical trial for ARDS/ALI (NCT01597635)	APN01-COVID-19 (NCT04335136) Bacterial ACE2 (NCT04375046)

Similarly, in the case it is confirmed that DPP4 plays a role in SARS-CoV-2 infection as co-receptor, the use of sDPP4 as decoy receptor could also be explored. However, in obesity and T2DM sDPP4 levels are upregulated, sDPP4-induced immunomodulatory actions might be altered and there is a reduced bioavailability of beneficial substrates, facing COVID-19 CV consequences might be a difficult task. Other potential therapeutic approaches worth exploring are DPP4i in order to determine if they can block virus entry, as described for sitagliptin in MERS. Irregardless of DPP4 role as co-receptor, gliptins might help prevent CV complications in COVID-19 due to their anti-inflammatory effects at vascular level. Anti-DPP4 vaccine has been proven effective in mice to control glucose homeostasis and might represent another potential approach.

In conclusion, further preclinical research and clinical data will help to better understanding of the SARS-CoV-2 virus (**Table 2**), and will allow to determine if any of these potential therapeutic strategies is useful.

**Table 2:** Summarized effects of sDPP4-targeted drugs (gliptins, sDPP4 and DPP4 vaccine) reported in preclinical (*in vivo* and *ex vivo*) and clinical research.

	Preclinical data		Previous clinical trials	COVID-19
	Cells	Animal models		Ongoing clinical trials
**Gliptins**	Block MERS-CoV infection in macrophages (Al-Qahtani et al., 2017) Prevention of sDPP4-induced hVSMC proliferation and inflammation (Wronkowitz et al., 2014)	Prevention of sDPP4-induced endothelial dysfunction in mice (Romacho et al., 2016) Anti-oxidant effects in STZ-induced diabetic rats and LPS-induced sepsis mouse model, respectively (Maeda et al., 2012; Beckers et al., 2017) Reduction of NLRP3/ASC inflammasome activation in db/db mice (Birnbaum et al., 2019) Improved cardiac function in mice (Picatoste et al., 2013)	Decreased risk of non-fatal CV events and CV mortality (Wang et al., 2017) No risk of infection in type 1 and type 2 diabetic patients (Yang et al., 2016) Increased cardio and vasculoprotective substrates (Brandt et al., 2006; Segers et al., 2011; Fadini et al., 2020) Anti-inflammatory effects in type 2 dibatec patients (Makdissi et al., 2012; Tremblay et al., 2014) Improved flow-mediated vasodilation in diabetic patients (Barchetta et al., 2019) Potential induction of leucopenia, angioedema, cough and asthma (Gosmanov and Fontenot, 2009; Baraniuk and Jamieson, 2010)	SIDIACO Sitagliptin (NCT04365517) Linagliptin: NCT04371978, NCT04341935
**sDPP4**	Block MERS-CoV infection in Vero cells (Raj et al., 2013) hVSMC proliferation and inflammation (Wronkowitz et al., 2014) Pro-inflammatory effects (Wronkowitz et al., 2014; Romacho et al., 2016) Inhibition of T-cell activation and proliferation *via* ADA binding (Zhong et al., 2013)	Endothelial dysfunction in murine mesenteric microvessels (Romacho et al., 2016) Increased monocyte migration in LDLR-/- mice (Sha et al., 2014)		Direct truncation of CCL5/RANTES: reduced IL-6 levels and viremia (Iwata et al., 1999; Patterson et al., 2020)
**Anti-DPP4 vaccine**	Blockade of MERS-CoV infection (Ohnuma et al., 2013) No effects on T-cell proliferation or cytokine production (Angevin et al., 2017)	Increased GLP-1 in type 1 and type 2 diabetic mice (Pang et al., 2014; Li et al., 2017)		

## Author Contributions

IV coordinated and wrote the manuscript. TR conceived, coordinated, and wrote the manuscript. CP, OL, CS-F, and JE wrote the manuscript. All authors discussed the main ideas and provided intellectual input of the complete version of the manuscript. All authors contributed to the article and approved the submitted version.

## Funding

IV is the recipient of a FPU fellowship (FPU16/02612). TR and JE are supported by KomIT-Center of Competence for Innovative Diabetes Therapy- funded by EFRE-NRW. CP and CS-F are supported by a grant from Plan Nacional de I+D (SAF2017-84776-R).

## Conflict of Interest

The authors declare that the research was conducted in the absence of any commercial or financial relationships that could be construed as a potential conflict of interest.

## Abbreviations

ACE2, angiotensin converting enzyme 2; ACEi, angiotensin converting enzyme 2 inhibitors; ALI, acute lung injury; Ang II, angiotensin II; Ang-(1-7), angiotensin-(1-7); ARB, angiotensin receptor 1 blockers; ARDS, acute respiratory distress syndrome; AT, anti-thrombin; ATR1, angiotensin receptor 1; ATR2, angiotensin receptor 2; BNP, brain natriuretic peptide; CCL5, chemokine (C-C Motif) ligand 5; COVID-19, coronavirus disease-19; CRP, C-reactive protein; CV, cardiovascular; DPP4, dipeptidyl peptidase 4; DPP4i, dipeptidyl peptidase 4 inhibitors; GIP, gastric inhibitory peptide; GLP-1, glucagon-like peptide-1; IL, interleukin; ICU, intensive care unit; LDLR, low-density-lipoprotein receptor; MERS, Middle East respiratory syndrome; Mrc1, macrophage mannose receptor 1; NO, nitric oxide; NT-pro-BNP, N-terminal pro-brain natriuretic peptide; PT, pro-thrombin; RAAS, renin-angiotensin aldosterone system; RANTES, regulated upon activation, normal T cell expressed and presumably secreted; RBD, receptor binding domain; SARS-CoV-2, severe acute respiratory syndrome coronavirus-2; STZ, streptozotocin; rhACE2, recombinant human angiotensin converting enzyme 2; sDPP4, soluble dipeptidyl peptidase 4; T2DM, type 2 diabetes mellitus; TnT, troponin T; VCAM-1, vascular cell adhesion molecule-1.
